# Measurement Structures of Image Compressive Sensing for Green Internet of Things (IoT)

**DOI:** 10.3390/s19010102

**Published:** 2018-12-29

**Authors:** Ran Li, Xiaomeng Duan, Yanling Li

**Affiliations:** School of Computer and Information Technology, Xinyang Normal University, Xinyang 464000, China; dxmLily@163.com (X.D.); liyanling@xynu.edu.cn (Y.L.)

**Keywords:** image compressive sensing (CS), green internet of things (IoT), measurement structure, random structural matrices, linear recovery

## Abstract

Image compressive sensing (CS) is a potential imaging scheme for green internet of things (IoT). To further make CS-based sensor adaptable to low bandwidth and low power, this paper focuses on finding a good measurement structure, i.e., the organization and storage format of CS measurements. Three potential measurement structures are proposed in this paper, respectively raster structure (RA), patch structure, and layer structure (LA). RA stores CS measurements of each column in an image, and PA packets CS measurements of overlapping patches forming an image. LA enables the measuring of small blocks and recovery of large blocks. All of the three structures avoid high computation complexity and huge memory in the process of measuring and recovery, and efficiently suppress the annoying blocking artifacts which often occur in traditional block structures. Experimental results show that RA, PA, and LA can efficiently reduce blocking artifacts, and produce comforting visual qualities. LA, especially, presents both good time-distortion and rate-distortion performance. By this paper, it is proved that LA is a suitable measurement structure for green IoT.

## 1. Introduction

### 1.1. Motivation and Objective

Up to this day, compressive sensing (CS) [1,2] has already become a commonplace technology, and it is widely used in imaging applications to sample various signals, e.g., magnetic resonance imaging (MRI) [3], multispectral imaging [4], synthetic aperture radar (SAR) imaging [5], etc. Moreover, in the field of microwave tomography and antenna synthesis, CS also becomes a popular tool, e.g., [6,7] use CS to solve the non-linear inverse scattering problems, and [8,9] use CS to solve power synthesis of maximally-sparse arrays. These applications reflect the low cost of CS in capturing invisible light which brings a cheap and portable sensor. Many researchers recognize that CS is an energy-efficient sampling method which provides a good imaging quality at the same time, so they have turned their attention to the application of CS to green internet of things (green IoT) [10,11].

To achieve green IoT, some frameworks [12,13,14] of image CS are reviewed and newly developed to meet the demand for these energy-hungry devices. These works focus on how to design sampling and recovery algorithms to get better imaging quality in low-rate and low-power cases, but they have not considered how to organize and store CS measurements to improve the efficiency of sensors in performing CS. In green IoT, measurement structure, i.e., the organization and storage format of CS measurements, is important for sensors to produce a compact bitstream that could reduce the energy consumption of transmission. Therefore, the objective of this paper is to find a good measurement structure to make CS-based sensors energy-efficient.

### 1.2. Related Work

In existing works, two kinds of measurement structures are used to organize CS measurements, i.e., whole structure (WH) and block structure (BL). WH regards whole image as a column vector, and measures it by using a random matrix. The early works [15,16,17,18] on image CS extensively applied WH in CS measuring, and they often spent much time in recovering a small size image by some complex numeric iterations. The limitation of these works results from the difficulty in constructing some excellent matrices, e.g., i.i.d. Gaussian matrix, under WH. When enlarging the image size, WH leads to high computation complexity for measuring and huge memory for storage, e.g., for an image of 512 × 512 in size, 512 gigabytes storage is required to construct a random matrix with entries being 64-bit floating points. This is impractical especially for low-power sensors in green IoT. To make WH suitable for large-scale image, some memory-friendly structural matrices were proposed, in which the pioneering work is structurally random matrix (SRM) proposed by Do et al. [19]. SRM replaces the matrix entries with operators of scrambling, fast transforming and sub-sampling, and it enables WH to fast encode a large-scale image while providing a good recovery quality. Based on SRM, some works added some special operators to construct better structural matrices, e.g., Zhang et al. [20] used a unit-norm tight frame as a part of SRM, and Hsieh et al. [21] used sparse FFT as the core of SRM. Two defects still exist in SRM-based WH. First, CS measurements are only encoded by scalar quantization (SQ) which, however, does not perform well in rate-distortion performance due to the randomness of measurements. Second, SRM enforces the recovery algorithm to perform matrix-vector product in the form of function handle which, however, are not supported by some popular recovery algorithms. These defects limit the wide use of WH in practice, so some researchers turn their interest to the structure BL.

BL splits a whole image into non-overlapping blocks and then regards a block as a column vector and finally, measures it by using a random matrix. Because blocks are small in size, the unstructured matrix like i.i.d. Gaussian matrix can be used, without the worry of high computation complexity and huge memory. This makes BL practical especially when sensors have limited power and computation resource in green IoT. Many works focus on BL-based sampling, quantization, and recovery methods, e.g., Gan [22] and Mun et al. [23] proposed the smoothed projected Landweber (SPL) algorithm to recover blocks; Yu et al. [24] and Zhang et al. [25] proposed to adaptively measure blocks according to their features; Mun et al. [26], Wang et al. [27], Dinh et al. [28], and Gao et al. [29] proposed various predictive schemes to quantize block measurements. These works win BL more popularity than WH in image CS. However, blocks are different in sparse degree in a fixed space, thus they vary in recovery quality when the same algorithm is performed, which leads to blocking artifacts. The challenge for BL is to suppress the annoying blocking artifacts. The existing works try to overcome this defect by designing sampling and recovery algorithms, but they neglect the fact that BL is the root cause of blocking artifacts. Therefore, we need to find other potential structures to make up for this deficiency of BL.

### 1.3. Main Contribution

This paper presents three potential measurement structures—raster structure (RA), patch structure (PA), and layered structure (LA)—and they can effectively reduce the blocking artifacts in BL. Compared with WH, these structures have lower power and rate, especially for RA and LA, so they are suitable to be used in sensors of green IoT. We carefully analyze the time-distortion and rate-distortion performance of image CS when deploying various structures, and conclude that LA is the most potential structure among them. Combining LA with linear recovery, CS-based sensor will be more suitable for green IoT.

The rest of this paper is organized as follows. Section 2 briefly describes the two traditional structures: WH and BL. Section 3 presents the three potential structures: RA, PA, and LA. Experimental results are presented in Section 4, and we conclude this paper in Section 5. For easy understanding, the acronyms and notations in this paper are listed in Table 1.

## 2. Traditional Structures

### 2.1. Whole Structure

**Figure 1:**
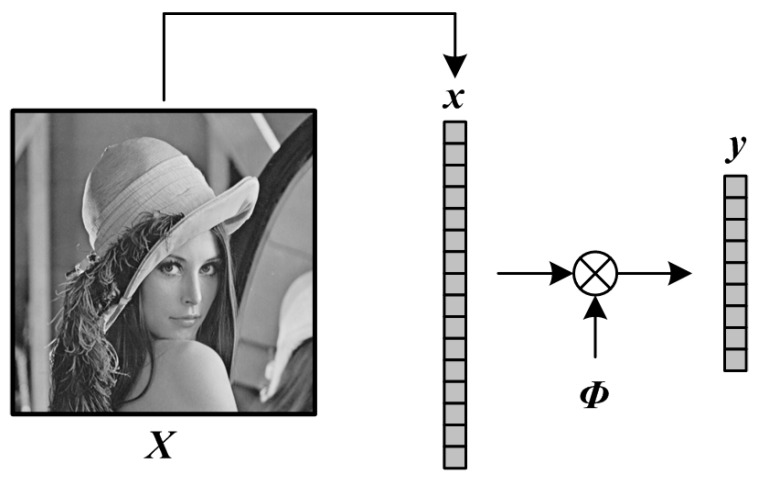
Illustration on WH format.

As shown in Figure 1, WH first transfers the 2-D image ***X*** ∈ ℝ*^I^*^c×*I*r^ (*N* = *I*_c_ × *I*_r_) to 1-D vector ***x*** ∈ ℝ*^N^*^×1^ through raster scanning, i.e.,

(1) x=Raster(X)

in which Raster(·) is an operator of raster scanning. Then, the measurement matrix ***Φ*** ∈ R*^M^*^×*N*^ is constructed, and we get the CS measurements ***y*** ∈ R*^M^*^×1^ of ***x*** as follows,

(2) y=Φ⋅x

in which *M* is the number of CS measurements, and the subrate *S* is defined as *M*/*N*. These CS measurements ***y*** are quantized by SQ to bits, and finally, these bits are packaged, and stored in the output buffering of sensor.

For a large-size image, limited by the memory size of sensor, it is impossible to construct the measurement matrix ***Φ***, so we cannot perform Equation (2). However, by SRM, we can get an operator equivalent to Equation (2) as follows,

(3) y=Φ(x)

in which ***Φ***(·) is a function handle of SRM. SRM is defined as a product of three matrices, i.e.,

(4) $$\Phi =\sqrt{\frac{N}{M}}DFR$$

in which ***R*** is a uniform random permutation matrix, ***F*** is an orthonormal matrix selected from some popular fast computable transforms, e.g., fast Fourier transform (FFT), discrete cosine transform (DCT), etc. ***D*** is a sub-sampling matrix, and $\sqrt{\frac{N}{M}}$ is to normalize the transform so that the energy of the output vector is almost similar to that of the input vector [19]. The three matrices have all the equivalent operators corresponding to their matrix-vector products, so a function handle of SRM can be designed to replace Equation (2). 

WH introduces a challenge for image recovery: the recovery algorithm must support the function handles of measurement and transform matrices. The WH-based recovery model is listed as

(5) $$\hat{\alpha }=\arg\underset{\alpha }{\min}{\left\|\alpha \right\|}_{0}\;s.t.\;y=\Phi (\Psi (\alpha ))$$

(6) $$\hat{x}=\Psi (\hat{\alpha })$$

in which ||·|| is *l*_0_ norm, ***Φ***(·) is a handle of SRM, and ***Ψ***(·) is a fast transform operator, e.g., DCT, FFT, etc. The existence of ***Φ***(·) and ***Ψ***(·) requires that ***Φ·x***, ***Φ***^T^***·x***, ***Ψ·x***, and ***Ψ***^T^***·x*** have the equivalent function handles (the superscript T represents transposition). From the above, it can be seen that WH requires the recovery algorithm to support function handle, so we only select some special algorithms, e.g., gradient projection for sparse reconstruction (GPSR) [15], to recover an image once from WH-based measurements.

### 2.2. Block Structure

**Figure 2:**
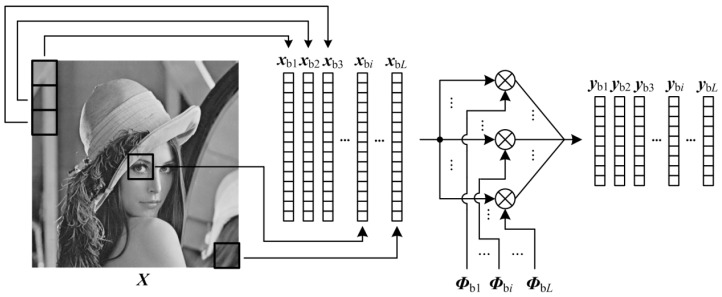
Illustration on BL format.

As shown in Figure 2, BL first splits the 2-D image ***X*** into *L* non-overlapping blocks ***X***_b*i*_ ∈ R*^B^*^×*B*^ (*N*_b_ = *B* × *B*) and transfer these blocks to 1-D vector ***x***_b*i*_ ∈ $R^{N_{b}\times 1}$ through raster scanning, i.e.,

(7) $$\left\{X_{bi}|i=1,2,\cdots ,L\right\}=\mathrm{Split}(X)$$

(8) $$x_{bi}=\mathrm{Raster}(X_{bi})$$

in which Split(·) is an operator of splitting image into non-overlapping blocks. Then, by constructing the block measurement matrix ***Φ***_b*i*_ ∈ $R^{M_{b}\times N_{b}}$, the CS measurements ***y***_b*i*_ ∈ $R^{M_{b}\times 1}$ of ***x***_b*i*_ are generated as

(9) $$y_{bi}=\Phi_{bi}\cdot x_{bi}$$

in which *M*_b_ is the number of CS measurements for each block, and the subrate *S* can be computed by *M*_b_/*N*_b_. Due to the small size of block, it requires a small memory size to construct the block measurement matrix ***Φ***_b*i*_, and the less computations are invested when performing Equation (9) block-by-block. These block measurements can be quantized to bits by the predictive quantization, e.g., DPCM [26], and finally, these bits are packaged block by block, and progressively stored in the output buffering of sensor. The predictive quantization shows better rate-distortion performance than SQ, so BL has a compact packet, which reduces the size of buffering in sensor.

BL has no special requirement on recovery algorithm. Each block can be reconstructed independently by any recovery algorithm, and especially, different from WH, a linear recovery method can be used to reconstruct blocks, i.e.,

(10) $${\hat{x}}_{bi}=\left\{R\cdot \Phi_{bi}^{T}\cdot {\left[\Phi_{bi}\cdot R\cdot \Phi_{bi}^{T}\right]}^{-1}\right\}\cdot y_{bi}$$

in which ***R*** is statistic auto-correlation matrix of ***x***_b*i*_, and its element *R_m_*_,*n*_ is approximated as follows,

(11) $$R_{m,n}={\left(0.95\right)}^{\delta_{m,n}}$$

in which *δ_m,n_* is the spatial distance between two pixels in ***x***_b*i*_, *x*_b*i*,*m*_ and *x*_b*i*,*n*_. Compared with conventional recovery algorithms, the linear recovery costs less computation while presenting a good recovery quality. Therefore, BL can both reduce the energy consumption of encoder and decoder, which is more suitable for green IoT. However, there are annoying blocking artifacts in BL-based recovery, so we need to explore potential structures to suppress blocking artifacts while preserving the advantages of BL at the same time.

## 3. Three Potential Structures

### 3.1. Raster Structure

**Figure 3:**
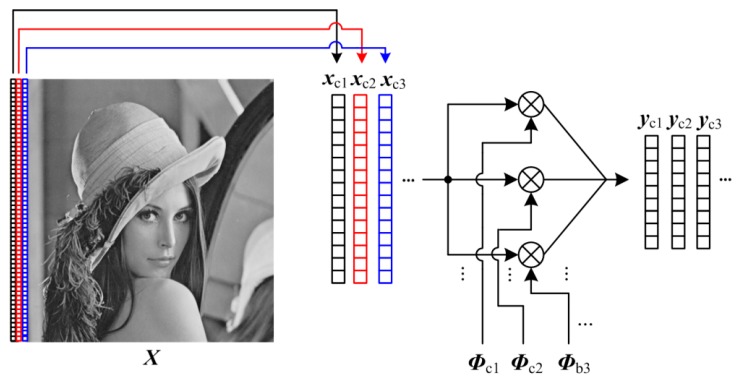
Illustration on RA format.

A key reason for blocking artifacts in BL is that the block is the basic unit of measuring and recovering. Blocking artifacts are gone once we irregularly spilt an image into sub-areas in other shapes. A simple way is to write the 2-D image in column form as

(12) $$X=\left[\begin{array}{cccc} | & | & & |\\ x_{c1} & x_{c2} & \cdots & x_{cI_{c}}\\ | & | & & | \end{array}\right]$$

in which ***x***_c*i*_ ∈ R*^I^*^r×1^ represents the *i*-th column vector. We can view these column vectors as the raster of image, so this segmentation is called raster structure (RA). As shown in Figure 3, these column vectors can be measured by a small-size matrix ***Φ***_c*i*_ ∈ R*^I^*^r×*I*r^, and the corresponding CS measurements ***y***_c*i*_ ∈ $R^{M_{c}\times 1}$ can be achieved as

(13) $$y_{ci}=\Phi_{ci}\cdot x_{ci}$$

in which *M*_c_ is the number of CS measurements for each column, and the subrate *S* can be computed by *M*_c_/*I*_r_. Because of high similarity between columns, the predictive quantization shows better rate-distortion performance for RA. Similar to BL, by replacing ***Φ***_b*i*_ and ***y***_b*i*_ in Equation (6) with ***Φ***_c*i*_ and ***y***_c*i*_, each column vector can be recovered by the linear algorithm, so RA retains the advantages of BL.

### 3.2. Patch Structure

**Figure 4:**
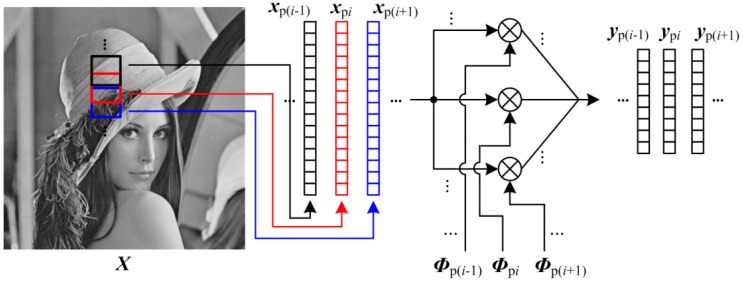
Illustration on PA format.

BL performs the recovery algorithm block by block, any two blocks are different in recovery quality because the statistical characteristic of block is non-stationary. Blocking artifacts are usually perceived in the regions where one block has significantly different statistics from its neighbors. By extending BL, we present a patch structure (PA) which split the 2-D image into overlapping patches. As shown in Figure 4, PA first splits the 2-D image ***X*** into *K* overlapping blocks ***X***_p*i*_ ∈ R*^P^*^×*P*^ (*N*_p_ = *P* × *P*), and transfer these patches to 1-D vector ***x***_p*i*_ ∈ $R^{N_{p}\times 1}$ through raster scanning. The size of overlap region between two neighboring patches is *P*/2, i.e., we slide a *P*×*P* patch in an image with the step size being *P*/2. By constructing the block measurement matrix ***Φ***_p*i*_ ∈ $R^{M_{p}\times N_{p}}$, the CS measurements ***y***_p*i*_ ∈ $R^{M_{p}\times 1}$ of ***x***_p*i*_ are generated as

(14) $$y_{pi}=\Phi_{pi}\cdot x_{pi}$$

in which *M*_p_ is the number of CS measurements for each patch, and the subrate *S* can be computed by *M*_p_/*N*_p_. The storage of ***Φ***_p*i*_ is moderate, so PA cannot introduce for sensors excessive burdens of computing and storing. By the predictive quantizing, these measurements are transferred to bits, and transmitted to decoder. By replacing ***Φ***_b*i*_ and ***y***_b*i*_ in Equation (6) with ***Φ***_p*i*_ and ***y***_p*i*_, the linear recovery algorithm is used to recover each patch, and these patches are spliced together into a whole image. PA smooths the boundary between two blocks, so it shows efficiency of reducing blocking artifacts.

### 3.3. Layered Structure

**Figure 5:**
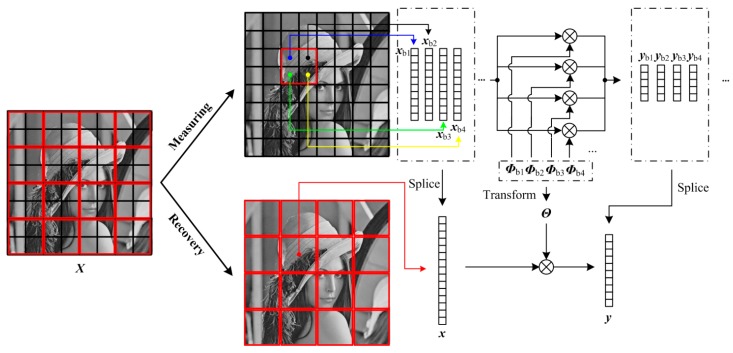
Illustration on LA format. The block-size pair is (*B*_1_, *B*_2_), and *B*_2_/*B*_1_ is 2.

Blocking artifacts results from the differences of neighboring blocks in recovery quality. The smaller the block size is, the more serious blocking artifacts are. The recovery quality is known to be better with a large block [22], so blocking artifacts can be effectively suppressed when we set a large block size in BL. However, considering the light burden of sensor, a small block size is more desired. Especially for predictive quantization, a high spatial correlation exists among small-size blocks, which would increase predicting efficiency of quantization. In view of that, as shown in Figure 5, we present the layered structure (LA) which can splice CS measurements of small blocks into those of large blocks, thus enabling the measuring of small blocks and the recovery of large blocks.

In LA, we set a block-size pair (*B*_1_, *B*_2_), in which *B*_1_ and *B*_2_ are the block size for measuring and recovery, respectively. To achieve measuring small block and recovering large block, *B*_1_ is smaller than *B*_2_, and they satisfy that,

(15) $$B_{2}=2^{l}\cdot B_{1}\;\begin{array}{c} l=1,2,\cdots \end{array}$$

where *l* is a positive integer. When performing CS measuring, as the same with BL, LA splits the 2-D image ***X*** into *L* non-overlapping blocks ***X***_b*i*_ ∈ $R^{B_{1}\times B_{1}}$ (*N*_b_ = *B*_1_ × *B*_1_) and transfer these blocks to 1-D vector ***x***_b*i*_ ∈ $R^{N_{b}\times 1}$ through raster scanning. By constructing the block measurement matrix ***Φ***_b*i*_ ∈ $R^{M_{b}\times N_{b}}$, the CS measurements ***y***_b*i*_ ∈ $R^{M_{b}\times 1}$ of these small blocks are produced, and we gather these CS measurements into the structure at the low layer. By re-organizing the low-layer structure, these CS measurements of small blocks are converted into those of large blocks which form the structure at high layer. The following describes the process of converting structure from low layer to high layer when *l* is set to be 1. As shown in Figure 5, each red block contains four black blocks which are measured as

(16) $$y_{bi}=\Phi_{bi}\cdot x_{bi}\begin{array}{cc} & i=1,2,3,4 \end{array}$$

and splice ***y***_b*i*_ into ***y*** in rows, i.e.,

(17) $$y=\left[\begin{array}{c} y_{b1}\\ y_{b2}\\ y_{b3}\\ y_{b4} \end{array}\right]=\left[\begin{array}{cccc} \Phi_{b1} & & & \\ & \Phi_{b2} & & \\ & & \Phi_{b3} & \\ & & & \Phi_{b4} \end{array}\right]\left[\begin{array}{c} x_{b1}\\ x_{b2}\\ x_{b3}\\ x_{b4} \end{array}\right]=\Phi_{\Lambda }\cdot \left[\begin{array}{c} x_{b1}\\ x_{b2}\\ x_{b3}\\ x_{b4} \end{array}\right]$$

in which ***Φ*** is the diagonal matrix composed of the block measurement matrices of four black blocks. These CS measurements of red blocks are gathered into the high-layer structure. However, from Equation (17), it can be seen that the column vector ***y*** does not correspond to the column vector ***x*** of the red block, so ***Φ***_Λ_ needs to be transformed into a proper matrix. By an elementary column transformation, it is easy to transform [***x***_b1_; ***x***_b2_; ***x***_b3_; ***x***_b4_] into ***x***, which can be represented as

(18) $$\left[\begin{array}{c} x_{b1}\\ x_{b2}\\ x_{b3}\\ x_{b4} \end{array}\right]=E\cdot x$$

in which ***E*** is an elementary column transformation matrix. Plugging Equation (18) into Equation (17), we can get

(19) $$y=\left[\begin{array}{c} y_{b1}\\ y_{b2}\\ y_{b3}\\ y_{b4} \end{array}\right]=\Phi_{\Lambda }\cdot \left[\begin{array}{c} x_{b1}\\ x_{b2}\\ x_{b3}\\ x_{b4} \end{array}\right]=\Phi_{\Lambda }E\cdot x=\Theta \cdot x$$

in which ***Θ*** = ***Φ***_Λ_***E***. According to Equation (19), we construct CS measuring formula of the red block, so we can use the CS measurements of black blocks to linearly recover the red ones by Equation (6). When *l* is set to be larger than 1, the construction of LA can be done in the manner similar to the above.

## 4. Experimental Results

In this section, we subjectively and objectively evaluate the reconstruction quality of some 512 × 512 test images using different measurement structures in image CS. These test images include *Lenna*, *Barbara*, *Peppers*, *Goldhill*, and *Mandrill*. In all experiments, the subrate *S* is set to be in the range of [0.1, 0.5]. For WH, DCT-based SRM [19] is used to construct the measurement matrix, SQ is used to quantize CS measurements, and GPSR algorithm is used to recover images. For BL, RA, PA, and LA, to ensure a fair comparison, i.i.d. Gaussian matrix is selected to be measurement matrix, DPCM is used to quantize CS measurements, and images are recovered linearly. The block size is set to be 16 in BL, and LA uses different block-size pairs in different experiments. Subjective evaluation shows the reconstructed images at different subrates using different structures. In objective evaluation, we measure the recovery quality in terms of peak signal-to-noise ratio (PSNR), bitrate, and encoding time. The variation of PSNR with bitrate is called rate-distortion, and the variation of PSNR with encoding time is called time-distortion. Bitrate indicates the size of data transmitted by sensor, and encoding time gives an indication of the amount of energy expended while encoding, so rate-distortion reflects the transmission efficiency, and time-distortion the energy efficiency. All experiments are conducted under the following computer configuration: Intel(R) Core (TM) i7@3.30 GHz CPU, 8 GB RAM, Microsoft Windows 764 bits, and MATLAB Version 7.6.0.324 (R2008a).

### 4.1. Subjective Evaluation

**Figure 6:**
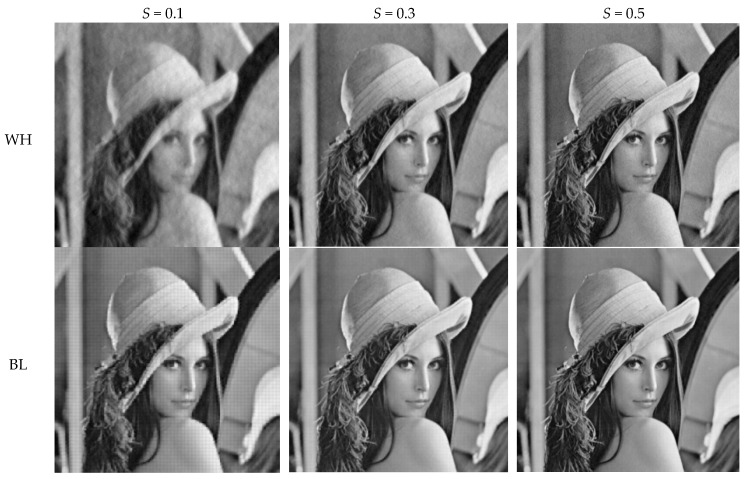

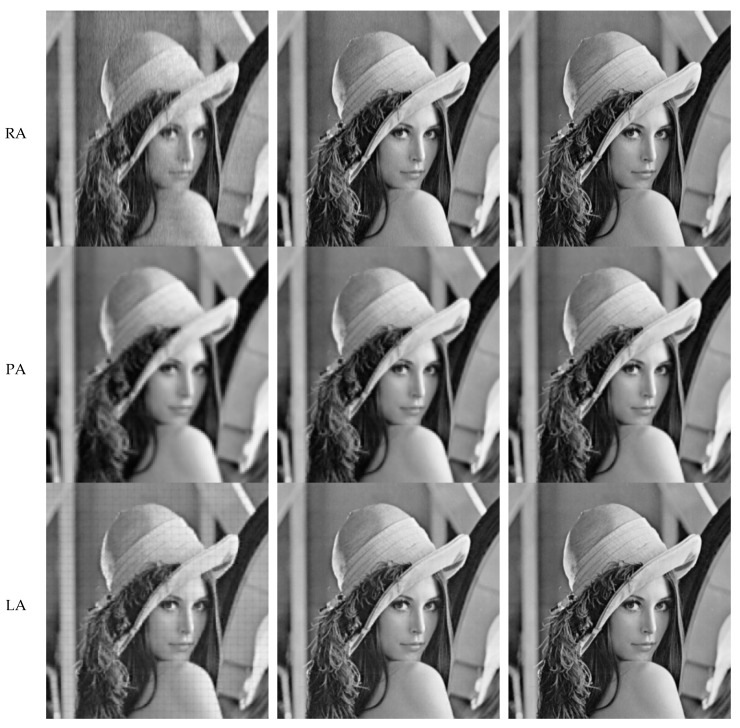
Visual quality comparison on test image Lenna for various measurement structures. Note: the block-size pair of LA is (8, 16).

**Figure 7:**
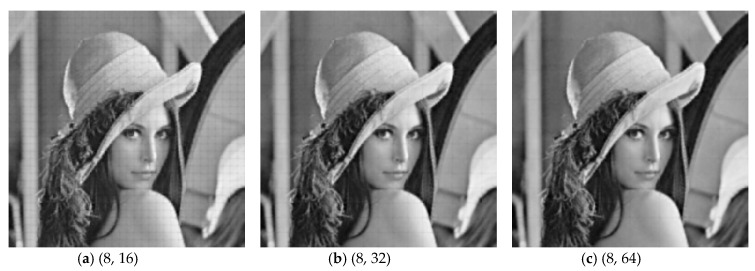

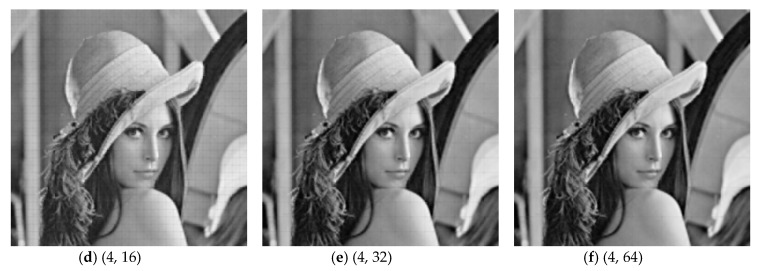
Visual quality comparison on test image Lenna for LA structures with various block-size pairs when the subrate is 0.1.

Figure 6 presents the visual recovery results of *Lenna* by various measurement structures at different subrates. When subrate *S* is 0.1, due to insufficient CS measurements, the reconstructed image by any structure lose structural details. It can be seen that, WH presented a disappointing result due to serious blurs, and BL bring lots of blocking artifacts. Although RA and PA can remove the blocking artifacts, they introduce other problems—e.g., obvious stripes in RA, global fuzzy in PA. When setting the block-size pair (8, 16), LA has a good visual perception when compared with BL, though many blocking artifacts still exist. When subrate *S* is increased to be 0.3, bad effects can be efficiently suppressed in WH, PA, and RA, and blocking artifacts almost disappear in BL and LA. When subrate *S* is 0.5, all structures provide satisfying visual results. we can see from the above that, none of the structures can guarantee good visual results when subrate *S* is 0.1, therefore, we expect to improve the visual quality at a low subrate. As shown in Figure 7, with a large *l*, LA can satisfy our expectation. When *l* is set to be 2, i.e., the block-size pair is (8, 32), blocking artifacts are reduced efficiently at subrate *S* = 0.1, and even when the block-size pair is (8, 64), blocking artifacts are almost imperceptible. When setting a small block size, as shown in Figure 7d–f, LA can still ensure a high efficiency for reducing blocking artifacts. Given the above, LA has greater potential than other structures from subjective view.

### 4.2. Objective Evaluation

**Figure 8:**
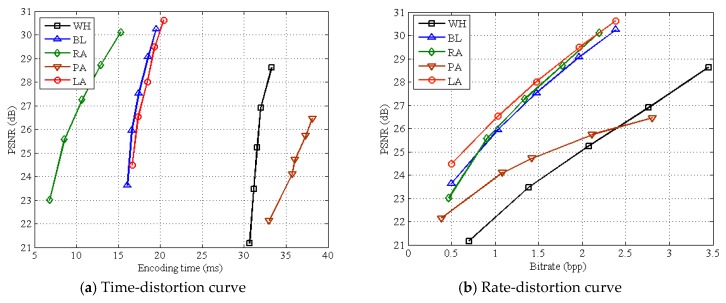
Average time-distortion and rate-distortion curves on test images for various measurement structures. Note: the block-size pair of LA is (8, 16).

**Figure 9:**
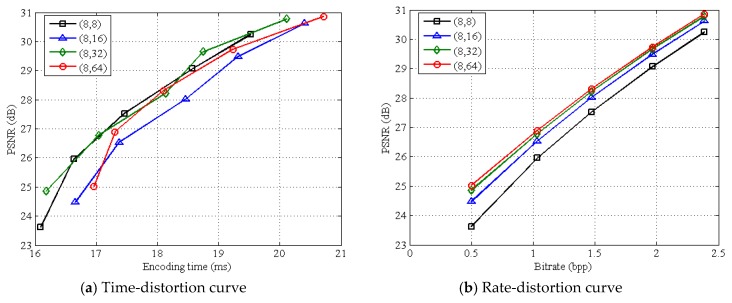
Average time-distortion and rate-distortion curves on test images for LA structures with various block-size pairs.

Table 2 presents the average encoding time of various structures on all test images when setting different subrates. WH and PA have a high execution time at any subrate, and their average time is respectively 31.74 and 36.05 ms on all subrates. BL, RA, and LA have a low execution time, among which RA does the best with only 10.86 ms on all subrates. Table 3 presents the average decoding time of various structures on all test images at different subrates. We can see that, the decoding time of WH is much longer than those of other structures, indicating that the non-linear recovery brings a heavy burden for WH. Due to linear recovery, BL, RA, PA, and LA all have a low decoding time, especially for LA, with only 6.03 ms at all subrates. Table 2 and Table 3 both considered, we can see that LA is a good structure for green IoT because it guarantees a low complexity at both encoder and decoder. Figure 8 shows average time-distortion and rate-distortion curves on test images for various measurement structures. It can be seen that, WH and PA have a poor time-distortion performance due to high encoding time, the time-distortion performance of BL is close to that of LA, and RA works the best among all structures. As for rate-distortion performance, WH and PA perform poorly, and LA works the best among all structures. Although RA has a good time-distortion performance, it produces more bits than LA. Figure 9 shows average time-distortion and rate-distortion curves on test images for LA structures with various block-size pairs. It can be seen that the rate-distortion performance can be significantly improved when block size *B*_1_ of measuring is much smaller than block size *B*_2_ of recovery, but different block-size pairs have little impact on time-distortion performance. To sum up, LA can save more bits while keeping both a low encoding time and a good recovery quality. Given the positive relation between encoding time and energy consumption, LA is the most suitable one among all structures for Green IoT from the objective view.

### 4.3. Effects of Measurement Matrices

In this section, we present the effects of different measurement matrices on the performance of BL, RA, PA, and LA. We select two classic measurement matrices, Gaussian matrix and permuted fast Fourier transform (PFFT) matrix [30], and two hardware-friendly matrices, ±Bernoulli matrix [31] and DCT-based SRM [19]. To ensure a fair comparison, all structures use linear algorithm to reconstruct test images. Table 4 presents the average encoding time of various structures on all test images at all subrates using different measurement matrices. It can be seen that, no matter which matrix is used, RA costs less encoding time than other structures, PA requires the most time among all structures, and BL and LA have a moderate encoding complexity. For any structure, the encoding time varies slightly even when different matrices are used. Table 5 presents the average PSNR values of various structures on all test images at all subrates using different measurement matrices. It can be seen that LA gets higher PSNR values than others regardless of matrix used. For any structure, Gaussian matrix provides higher PSNR values than others, and ±Bernoulli and SRM matrices have a little PSNR degradation compared with Gaussian matrix. We can find from the above that both encoding time and imaging quality considered, LA provides a good balance for any measurement matrix, and ±Bernoulli and SRM matrices have little effect on the performance of different structures compared with Gaussian matrix. In order to make LA hardware-friendly, therefore, Gaussian matrix can be replaced with ±Bernoulli and SRM matrices.

### 4.4. Effects of Recovery Algorithms

In this section, we present the effects of different recovery algorithms on the performance of different structures. All structures use DCT-based SRM to measure test images. The four recovery algorithms, namely GPSR, orthogonal matching pursuit (OMP) [32], Bayesian recovery [33], and linear recovery, are used to reconstruct test images. To ensure good performance of non-linear algorithms, the block size is set to be 32 for BL and PA, and the block-size pair of LA is set to be (32, 64). Table 6 presents the average decoding time of different structures on all test images at all subrates using different recovery algorithms. We can see that non-linear algorithms cost far more decoding time than linear algorithm for any structure, and LA costs more decoding time than BL, RA, PA, for linear algorithm, indicating that linear algorithm has a light computational burden compared with non-linear algorithms, and LA increases the decoding computation. Table 7 presents the average PSNR values of different structures on all test images at all subrates using different recovery algorithms. It can be seen that linear algorithm provides higher PSNR values than non-linear algorithms for any structure, especially that LA cooperated with linear recovery achieves the highest PSNR value. From the above, we can find that linear algorithm is more suitable for BL, RA, PA, and LA in terms of either computational complexity or objective quality, and LA can improve the recovery quality at the cost of some computational burden increase.

## 5. Conclusions

In this paper, we have reviewed two traditional measurement structures of image CS, i.e., WH and BL, and propose three potential structures for Green IoT, i.e., RA, PA, and LA. As a straightforward structure, WH stores CS measurements of a whole image at a time, but must use SRM to measure and recover image in order to avoid the huge storage and computations. BL organizes CS measurements block by block, with high energy efficiency and low storage, but it produces serious blocking artifacts at the low subrate. By replacing non-overlapping block in BL with raster and overlapping block, RA and PA can remove blocking artifacts, though bringing other problems. LA splits image into small blocks, and re-organizes these measurements of small blocks into those of large ones. Since small block improves the efficiency of predictive quantization, and large block enhances the recovery quality, LA can efficiently suppress blocking artifacts. We perform several experiments to evaluate the subjective and objective performances of all structures. At a low subrate, by setting a proper block-size pair, LA almost eliminates blocking artifacts, and provides better visual quality than other structures. It shows good time-distortion and rate-distortion performances, especially for rate-distortion performance, it can significantly improve it by setting a large block size of recovering. Both subjective and objective evaluation considered, it can be seen that LA is the most suitable for green IoT among structures.

This paper only presents an exploratory research, and there are many intriguing questions that future work should consider. For instance, for PA, a patch overlaps its neighbors, so we can design more efficient recovery algorithm by using the correlation among patches. Also, parallel computing can be adopted to speed up the linear recovery for LA.

## Figures and Tables

**Table 1:** List of acronyms and notations.

CS	Compressive Sensing
Green IoT	Green Internet of Things
WH	Whole Structure
BL	Block Structure
RA	Raster Structure
PA	Patch Structure
LA	Layered Structure
SQ	Scalar Quantization
DPCM	Differential Pulse Code Modulation
SRM	Structurally Random Matrix
DCT	Discrete Cosine Transform
FFT	Fast Fourier Transform
GPSR	Gradient Projection for Sparse Reconstruction
PSNR	Peak Signal-to-Noise Ratio

**Table 2:** Execution time (ms) of encoder for various measurement structures.

Subrate	0.1	0.2	0.3	0.4	0.5	Average
WH	30.68	31.17	31.57	31.97	33.28	31.74
BL	16.09	16.63	17.46	18.57	19.52	17.65
RA	6.82	8.57	10.69	12.91	15.31	10.86
PA	33.01	35.72	36.06	37.36	38.12	36.05
LA	16.66	17.37	18.46	19.31	20.41	18.44

Note: the block-size pair of LA is (8, 16).

**Table 3:** Execution time (ms) of decoder for various measurement structures.

Subrate	0.1	0.2	0.3	0.4	0.5	Average
WH	1.22 × 10^5^	1.00 × 10^5^	0.65 × 10^5^	0.52 × 10^5^	0.31 × 10^5^	0.74 × 10^5^
BL	10.12	10.21	10.16	10.14	10.33	10.19
RA	5.67	7.92	8.66	10.05	12.88	9.04
PA	21.18	21.71	21.92	21.72	21.59	21.62
LA	5.39	5.69	6.03	6.51	6.54	6.03

Note: the block-size pair of LA is (8, 16).

**Table 4:** Average execution time (ms) of encoder for different structures using different measurement matrices.

	Gaussian	PFFT	±Bernoulli	SRM
BL	17.65	18.35	18.14	18.17
RA	10.86	11.13	10.67	10.66
PA	36.05	34.23	33.11	33.28
LA	18.44	18.71	18.14	18.61

Note: the block-size pair of LA is (8, 16).

**Table 5:** Average PSNR values (dB) for different structures using different measurement matrices.

	Gaussian	PFFT	±Bernoulli	SRM
BL	27.28	25.70	26.88	26.69
RA	26.94	16.22	26.92	26.94
PA	24.63	24.22	24.38	24.24
LA	27.82	26.09	27.53	27.48

Note: the block-size pair of LA is (8, 16).

**Table 6:** Average execution time (ms) of decoder for different measurement structures using different recovery algorithms.

	GPSR	OMP	Bayesian	Linear
WH	7.44 × 10^4^	--	--	--
BL	9.34 × 10^4^	5.57 × 10^3^	6.26 × 10^4^	114.36
RA	9.00 × 10^4^	5.56 × 10^3^	6.64 × 10^4^	127.11
PA	1.14 × 10^5^	2.99 × 10^3^	7.73 × 10^4^	71.97
LA	5.32 × 10^5^	1.70 × 10^5^	2.14 × 10^5^	3.94 × 10^3^

Note: due to the unavailability of function handle, OMP, Bayesian and linear algorithms cannot be used for WH.

**Table 7:** Average PSNR values (dB) for different measurement structures using different recovery algorithms.

	GPSR	OMP	Bayesian	Linear
WH	25.08	--	--	--
BL	22.93	23.07	24.13	28.01
RA	19.80	20.63	21.63	26.94
PA	21.03	21.61	21.57	25.44
LA	23.04	23.32	24.47	28.12

Note: due to the unavailability of function handle, OMP, Bayesian and linear algorithms cannot be used for WH.
